# Clinician attitudes toward palliative care to enhance the quality of life for patients with sickle cell disease

**DOI:** 10.1093/jscdis/yoaf030

**Published:** 2025-09-16

**Authors:** Eberechi Nwogu-Onyemkpa, Amber B Amspoker, Nimrah Saleem, Aanand D Naik, Elizabeth Kvale, Ifeyinwa Osunkwo

**Affiliations:** Division of Palliative Care, Department of Medicine, Washington University in St Louis, St Louis, MO 63110, United States; Department of Medicine, Section of Health Sciences Research, Baylor College of Medicine, Houston, TX 77030, United States; VA Center of Innovations in Quality, Effectiveness and Safety, DeBakey VA Medical Center, Houston, TX 77030, United States; Department of Medicine, Section of Geriatrics and Palliative Medicine, Baylor College of Medicine, Houston, TX 77030, United States; VA Center of Innovations in Quality, Effectiveness and Safety, DeBakey VA Medical Center, Houston, TX 77030, United States; Department of Management, Policy, and Community Health, University of Texas School of Public Health & UTHealth Houston Institute on Aging, Houston, TX 77030, United States; Department of Medicine, Section of Geriatrics and Palliative Medicine, Baylor College of Medicine, Houston, TX 77030, United States; Novo Nordisk A/S Rare Disease, Zurich 8058, Switzerland; Maya Angelou Center for Health Disparities, Wake Forest University, Wake Forest, NC 27101, United States

**Keywords:** sickle cell disease, palliative care, quality of life, symptom management, supportive care, multidisciplinary care

## Abstract

**Objectives:**

Palliative care (PC) is rare for patients with sickle cell disease (SCD). This study evaluated clinician attitudes toward PC for SCD.

**Methods:**

A cross-sectional survey was conducted between October 2022 and February 2023. Clinicians were recruited from institutions across the United States.

**Results:**

Eighty-six participants completed the survey. Most were physicians (90%), female (72%), had >10 years of experience (63%), and lacked prior PC training (57%). Most participants agreed (83%) that patients with SCD would benefit from PC, disagreed (99%) that PC is only for dying patients, and expressed interest (91%) in learning about PC interventions. Clinicians with prior PC training were more likely to agree that patients with SCD would benefit from PC (*p *= 0.016). Most participants thought patients referred to PC would worry about discussions on dying (76%), might feel abandoned (63%), might feel greater control (59%), and would feel cared for by their SCD provider (65%). Survey responses indicated that 41% and 63% of participants had never worked with PC in a collaborative care inpatient or outpatient setting, respectively.

**Conclusion:**

Clinicians caring for patients with SCD recognize the potential benefits of PC, although they are unsure about patient responses to PC referrals. Collaborative care teams of SCD and PC specialists are rare. Educating clinicians and patients with SCD about PC is urgently needed to encourage greater collaboration among care teams, particularly in outpatient settings. Strengthened collaborations between SCD and PC specialists could enhance holistic support and quality of life for patients with SCD.

## INTRODUCTION

Sickle cell disease (SCD) is a genetic blood disorder affecting more than 100,000 persons in the United States, predominantly African Americans, and manifests as a complex multisystem condition with diverse symptoms.^1^ Complications of SCD arise directly from end-organ damage caused by recurrent ischemic events during vaso-occlusive episodes. These episodes lead to complications such as acute chest syndrome, severe anemia, acute stroke, recurrent infections, and priapism, which contribute to increased morbidity and mortality. Chronic organ damage from SCD also becomes prevalent over time and manifests as debilitating chronic pain and bone disease, chronic kidney disease and renal failure, pulmonary hypertension, heart failure, liver dysfunction, leg ulcers, and cognitive decline.^2^ Acute and chronic complications lead to high healthcare utilization in persons with SCD.^3–5^ Although most SCD patients now survive to adulthood,^6^ they continue to bear significant burdens of chronic comorbidities, which shorten the lifespan. Mean life expectancy is approximately 30 years less in those with SCD than in the general population.^7^ A recent study showed that more than 70% of individuals with SCD die in acute care settings, notably in hospitals or emergency rooms.^8^

Individuals with SCD experience substantial physical and psychological burdens including acute and chronic pain,^9^^,^^10^ fatigue,^11^ and increased prevalence of depression and anxiety.^12^ These symptoms often are not optimally managed, which leads to profound impacts on daily life.^11^ These challenges are further compounded by the systemic racism and discrimination that patients frequently encounter when seeking medical care.^13^ Unfortunately, many patients, especially adults with SCD, lack access to comprehensive care teams or medical homes that can meet their complex medical and psychosocial needs.^14^^,^^15^ Consequently, children and adults with SCD report lower health-related quality of life than the general population and those with other chronic diseases such as cystic fibrosis (CF).^16^^,^^17^ An international study with a large cohort of individuals with SCD reported that the most common treatment goal was to improve the quality of life.^11^

Palliative care (PC) uses an interdisciplinary approach to improve the quality of life of patients and families facing serious illnesses. There has been significant uptake of PC in oncology,^18^^,^^19^ but other specialist disciplines (eg, hematology) have been slower to integrate PC into care plans for individuals with chronic illnesses. Prior studies on barriers to PC integration in hematology focused on hematologic malignancies and not on benign conditions like SCD.^20^^,^^21^ Clinician-specific barriers to PC integration in malignant hematology include misperceptions that PC is only for end-of-life care, a lack of knowledge about the full scope of PC interventions, a strong sense of ownership over managing the needs of their patients, and the belief that a PC referral would not be well-received and would reduce hope in patients and families.^20^^,^^22^^,^^23^ PC could potentially alleviate suffering and reduce healthcare utilization in SCD. However, the current use of specialist PC services in SCD is low; only 0.45% of SCD-related hospitalizations receive PC, and this referral typically occurs late in the disease course.^24^ To date, no studies have evaluated barriers to PC integration in SCD, although clinician attitudes and perceptions may contribute to the low use of specialist PC interventions in SCD.

The objective of this study is to evaluate clinician attitudes toward PC for their patients with SCD, and determine clinician perceptions of how patients would react to a PC referral. We also examined clinician awareness of PC, the frequency of their collaboration with PC teams, and their direct involvement in and sense of ownership of PC interventions. We also performed stratified analyses to investigate potential correlations between clinician demographics and their attitudes toward PC. We hypothesize that clinicians with prior PC training and experience will likely report more positive attitudes toward PC than providers of SCD care who lack prior PC education or collaboration.

## MATERIALS AND METHODS

### Study population and recruitment

**Figure 1.**
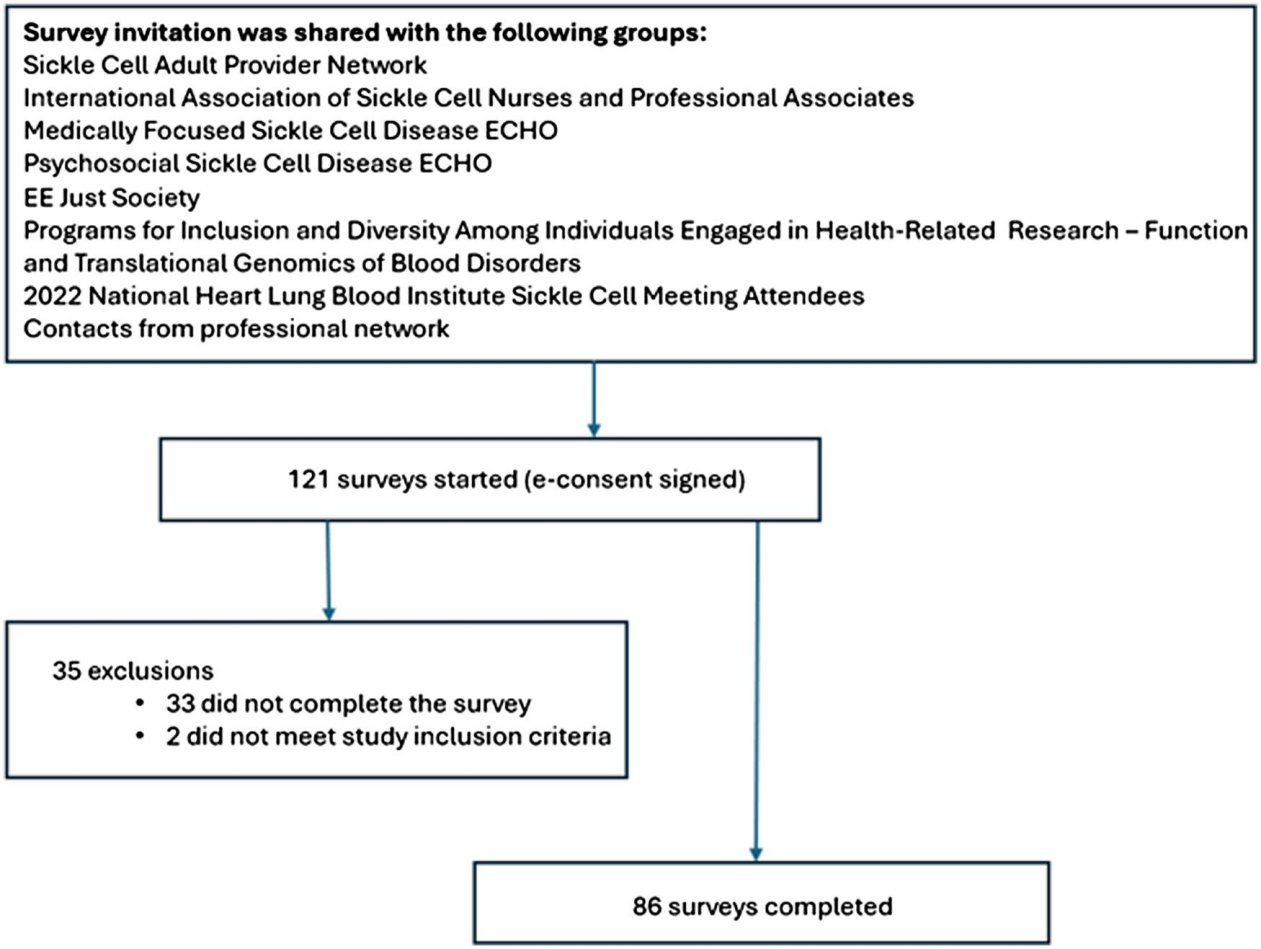
Study design and participant recruitment. List of SCD provider groups that were contacted to recruit study participants and number of surveys started and completed.

The study inclusion criteria were US clinicians treating SCD in adult and pediatric patients, whereas exclusion criteria were current clinical practice that did not include the care of adult or pediatric patients with SCD. A screening question was used to identify participants who did not meet the study inclusion criteria (“How much of your practice involves the care of patients with SCD?”). Clinicians were required to be treating at least one patient with SCD to be included in the study. We aimed to include 100 US clinicians who provide direct clinical care and manage pediatric and/or adult patients with SCD. There is no central listing of providers treating SCD; therefore, we targeted specific provider groups treating SCD (Figure 1). We were aware that some potential participants could belong to multiple provider groups, and every member of these groups would not meet the study inclusion criteria. However, our targeting strategy should successfully recruit a heterogeneous group of providers, including physicians, nurse practitioners, and physician assistants treating SCD across multiple medical specialties, including hematology, oncology, internal medicine, pediatrics, and family medicine. We sent an email containing the study objective and a link to the survey to members of the groups. We also used the snowball recruitment method to contact additional clinicians within the authors’ professional networks. In addition, the survey was advertised on social media platforms including LinkedIn and X (formerly Twitter).

### Survey design and development

A survey was developed to assess clinician attitudes toward PC and their perceptions of patient responses to PC referral in SCD. The survey was created by reviewing and adapting relevant questions from previous surveys that evaluated attitudes and perceptions toward PC among oncologists, hepatologists, and gastroenterologists.^15^^,^^16^ We removed questions that were not relevant for SCD and added new questions that were aligned with our study objectives. The survey was reviewed by two SCD and two PC content-expert physicians who provided feedback on the survey clarity. The survey was revised based on the feedback of these four physician reviewers. The final survey contained 60 items and is available as a supplemental file. This study was approved by the Institutional Research Board of Baylor College of Medicine.

The survey assessed clinician responses in the following six domains: (1) attitudes about PC (7 items), (2) awareness of PC (2 items), (3) collaboration with PC and other interdisciplinary teams (9 items), (4) direct involvement in PC interventions (13 items), (5) sense of ownership over PC interventions (7 items), and (6) perception of their patient reactions to PC referral (9 items). The survey used two three-point Likert scales: the response options of the first scale were “never,” “occasionally,” and “often,” whereas the response options of the second scale were “rarely,” “occasionally,” and “often.” The survey also used a four-point Likert scale with response options of “strongly disagree,” “disagree,” “agree,” and “strongly agree.”

The survey also recorded the demographic characteristics of participants including age, sex, terminal degree, medical specialty, years of clinical practice, clinical practice setting, practice type, patient population served, prior PC training or exposure, and prior history of referrals to PC.

### Survey administration

The survey was administered via the web-based Research Electronic Data Capture (REDCap, Vanderbilt University, Nashville, TN, United States) platform. Members of the selected study groups received invitations to participate in the survey via an email containing the link to the web-based survey. Follow-up emails were sent to the groups to recruit additional participants, although it was not feasible to track individual providers who completed the survey. The survey was distributed between October 2022 and February 2023.

### Statistical analysis

Participant responses to the 60 survey items representing six domains were analyzed using descriptive statistics, including frequencies and percentages. Subsequent analyses examined differences in percent agreement (“agree” vs “strongly agree”) regarding clinician attitudes about PC and their perceptions of patient responses to PC referral, and these differences also were evaluated for potential correlations with participant demographic characteristics. We used Fisher’s exact test and considered a *P*-value < .05 as statistically significant. All analyses were conducted using SAS version 9.4 (SAS Institute, Cary, NC, United States).

## Results

### Participant characteristics

A total of 86 participants completed the survey. The demographic characteristics of the study participants are presented in Table 1. Most participants were physicians (90%), female (72%), and had more than 10 years of clinical experience (63%). Most participants (79%) reported that more than 25% of their clinical practice involved care for patients with SCD. Most participants did not have prior PC training (57%). More than half of participants reported that they had previously referred a patient with SCD to PC (59%).

**Table 1.** Demographic characteristics of study participants.

Participant characteristics	Number of study participants (%) Total, *N *= 86 participants
**Sex**	
Cis-Female	62 (72)
Cis-Male	21 (24)
Nonbinary/non-confirming/gender-fluid	1 (1)
Prefer not to respond	2 (2)
**Age**	
30-39	25 (29)
40-49	32 (37)
50-59	11 (13)
>60	18 (21)
**Degree**	
MD or DO	77 (90)
Nurse practitioner (NP)	8 (9)
Other	1 (1)
**Medical specialty**	
Adult hematology	35 (41)
Pediatric hematology	38 (44)
Internal medicine	4 (5)
Family medicine	3 (3)
Other	6 (7)
**Patient population**	
Pediatric	15 (17)
Adult (>18 years)	43 (50)
Combined pediatric and adult	28 (33)
**Practice type**	
Private hematology practice	1 (1)
Community hospital-based	6 (7)
Teaching hospital-based	40 (47)
Comprehensive SCD center	38 (44)
Other	1 (1)
**Practice setting**	
Inpatient	6 (7)
Outpatient	13 (15)
Combined inpatient and outpatient	67 (78)
**How much of your practice involves care of patients with SCD?**	
Less than 25%	17 (20)
Greater than 25%	15 (17)
Greater than 50%	54 (63)
**Years of clinical experience**	
<5	14 (16)
5–9	17 (20)
10–14	16 (19)
15–19	13 (15)
20–24	6 (7)
>25	20 (23)
**Prior palliative care training**	
None	49 (57)
Attended PC courses, CME lectures	23 (27)
Formal PC rotation during residency or fellowship	12 (14)
≥ 6 months of formal PC training	2 (2)
**Have you ever referred a patient with SCD to a PC specialist or team?**	
No	35 (41)
Yes	51 (59)

Abbreviation: CME, continuing medical education.

### Clinician awareness and attitudes toward palliative care

**Figure 2.**
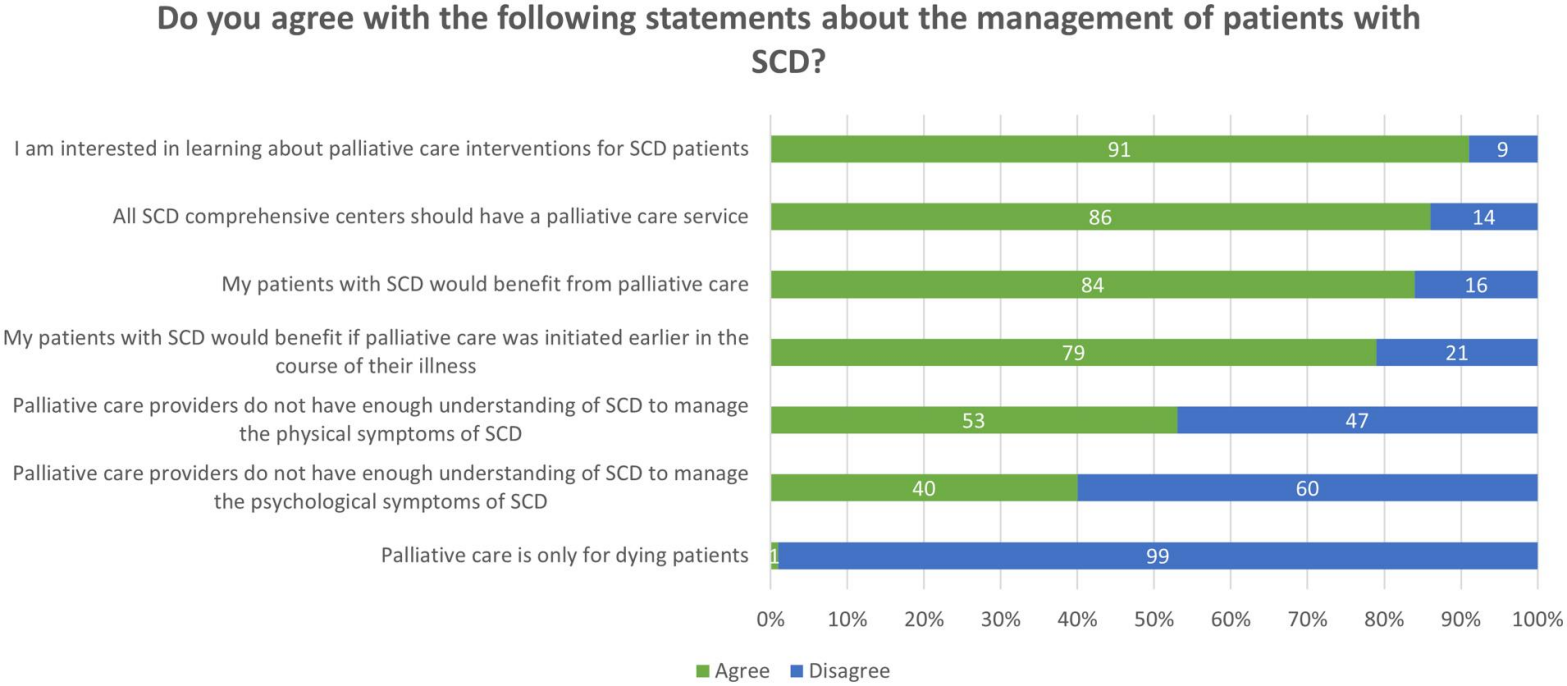
Participant responses to survey items regarding SCD clinician attitudes about palliative care. “Agree” and “strongly agree” responses were combined. “Disagree” and “strongly disagree” responses were combined. The values represent percent of participant responses.

Most clinicians reported that they were aware of the interventions that PC teams can provide for their patients with SCD (77%), and knew how to refer their patients with SCD to PC (74%). Participant responses to survey items regarding clinician attitudes toward PC are presented in Figure 2. Most clinicians agreed that patients with SCD would benefit from PC (83%), particularly from early initiation of PC (79%).

We examined differences in positive attitudes toward PC stratified by clinician demographic characteristics (Table 2). Clinicians who reported prior referral to PC were significantly more likely to agree that patients with SCD would benefit from PC than those who reported no prior PC referral experience (92% vs 71%, respectively; Fisher’s exact test, *P *= .016). Clinicians with prior PC training were more likely to respond positively to the three PC attitude items than those without prior PC training, although this difference was not statistically significant. Female participants were more likely to agree that patients with SCD would benefit from early PC initiation than male participants (84% vs 62%, respectively). Clinicians with less than 5 years and 5-14 years of clinical practice were more likely to agree that patients with SCD would benefit from PC than those with more than 15 years of clinical practice (93% vs 91% vs 74%, respectively).

**Table 2.** Differences in percent agreement with survey items regarding SCD clinician attitudes toward palliative care, stratified by demographic characteristics.

	My patients with SCD would benefit from palliative care		My patients with SCD would benefit if palliative care was initiated earlier in the course of their illness		All SCD comprehensive centers should have a palliative care service	
	*N* (%)	*P*-value^a^	*N* (%)	*P*-value^a^	*N* (%)	*P*-value^a^
**Sex**						
Cis-Female	52 (84%)	.74	52 (84%)	.06	54 (87 %)	.49
Cis-Male	17 (81%)		13 (62%)		17 (81%)	
**Medical specialty**						
Adult hematology	31 (89%)	.35	28 (80%)	1.00	29 (83%)	.75
Pediatric hematology	30 (79%)		30 (79%)		33 (87%)	
**Years of clinical practice**						
<5 years	13 (93%)	.16	11 (79%)	.60	13 (93%)	.37
5-14 years	30 (91%)		28 (85%)		30 (91%)	
≥15 years	29 (74%)		29 (74%)		31 (79%)	
**Prior palliative care training**						
No	39 (80%)	.38	37 (76%)	.43	41 (84%)	.54
Yes	33 (89%)		31 (84%)		33 (89%)	
**Practice type**						
Teaching hospital	33 (83%)	1.00	30 (75%)	.59	35 (88%)	.75
Comp SCD center	32 (84%)		31 (82%)		32 (84%)	
**Prior referral to palliative care**						
No (*n *= 35)	25 (71%)	**.016**	25 (71%)	.18	30 (88%)	1.00
Yes (*n *= 51)	47 (92%)		43 (84%)		44 (86%)	

The total number of participants who agreed (“agree” and “strongly agree” responses were combined) with the survey item is presented as *N* (%). Bold indicates statistically significant value.

a Fisher’s exact test was used to calculate *P*-values.

Abbreviation: Comp SCD center, comprehensive sickle cell disease center.

Essentially all respondents (99%) disagreed that PC is only for dying patients, and 91% were interested in learning more about PC interventions in SCD. Some respondents believed that PC specialists do not sufficiently understand SCD to manage its physical (52%) and psychological (41%) symptoms.

### Direct involvement of clinicians in PC interventions

**Figure 3.**
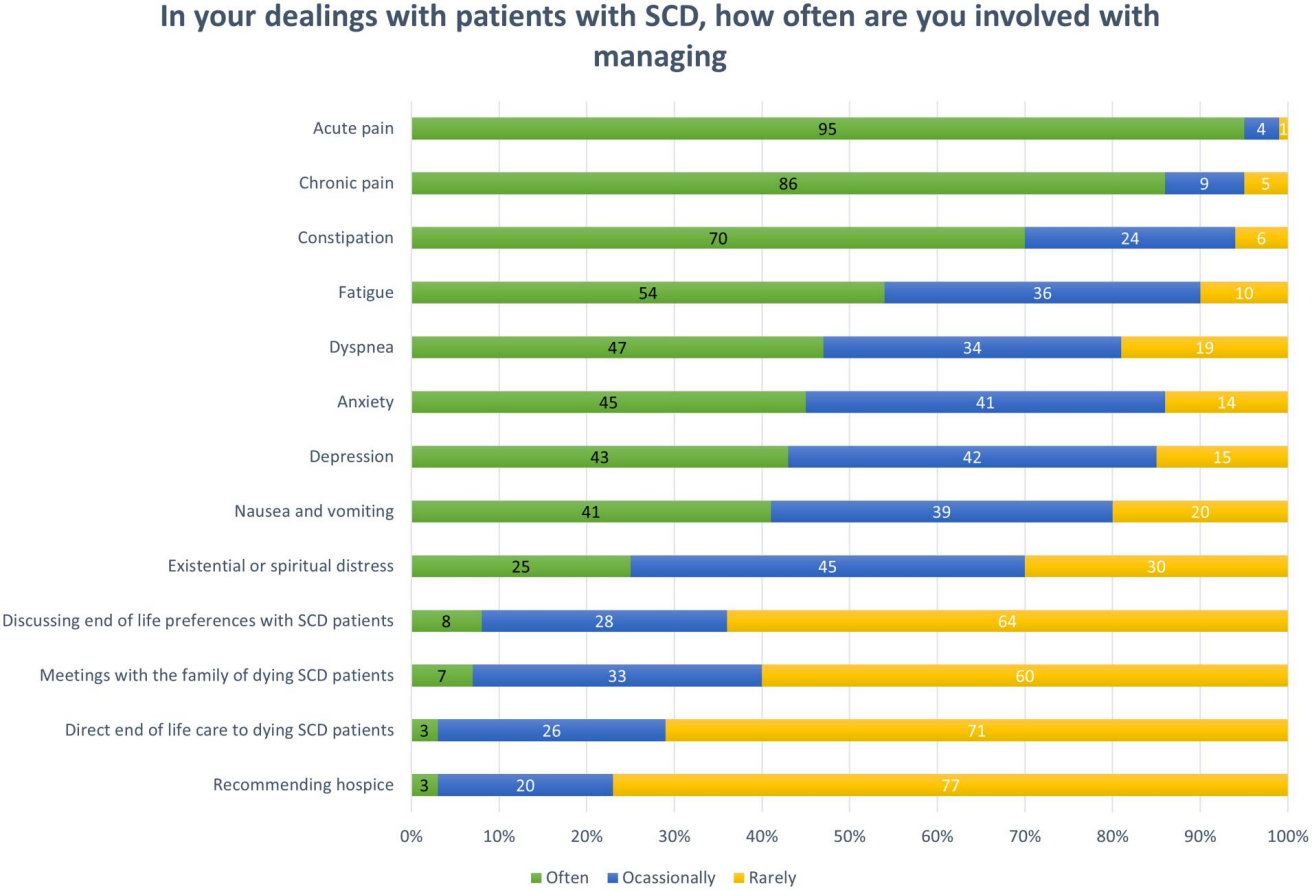
Participant responses to survey items regarding SCD clinician involvement in palliative care interventions. The values represent percent of participant responses.

Participant reports of direct involvement in managing a range of PC interventions are presented in Figure 3. Essentially all providers reported frequent or occasional involvement in managing acute pain (99%) and chronic pain (95%). Most providers reported frequent or occasional involvement in managing constipation (94%), fatigue (90%), anxiety (86%), depression (85%), dyspnea (81%), nausea/vomiting (80%), and existential and spiritual distress (70%). By contrast, many providers reported that they were rarely involved in discussing end-of-life preferences with SCD patients (64%), coordinating meetings with the family of dying SCD patients (60%), providing end-of-life care to dying SCD patients (71%), or recommending hospice for SCD patients (77%).

### Sense of ownership of PC interventions

**Figure 4.**
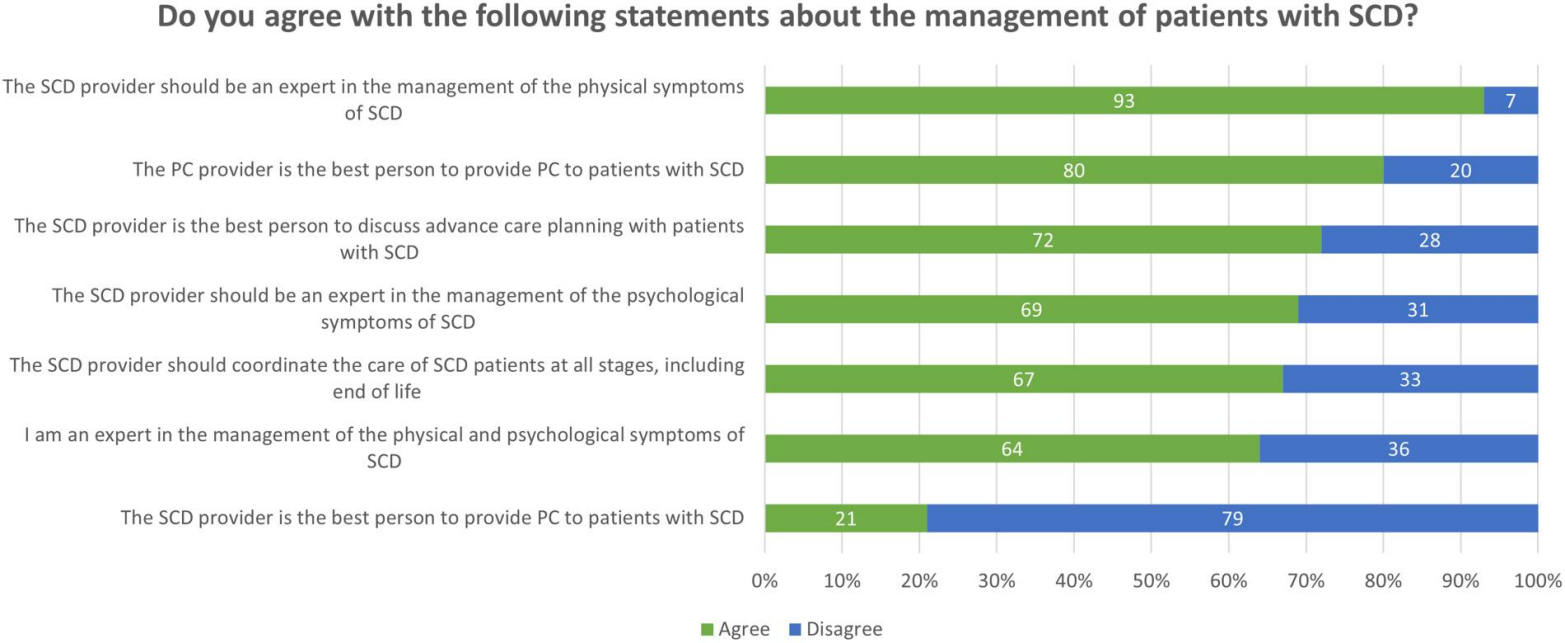
Participant responses to survey items regarding the frequency of SCD clinician collaboration with palliative care clinicians and other interdisciplinary teams. The values represent percent of participant responses.

Participant responses to items regarding a sense of ownership of PC interventions for their SCD patients are presented in Figure 4. Most participants agreed that the SCD provider should have expertise in managing the physical (93%) and psychological (69%) symptoms of SCD, and should coordinate the care plan at all stages, including end-of-life (67%). More than half of participants (64%) considered themselves expert in managing SCD symptoms, although most (79%) disagreed that the primary clinician has the best specialist expertise to provide PC to patients with SCD. Most participants (80%) agreed that the PC clinician is the best specialist to provide PC to patients with SCD.

### Clinician collaboration with palliative care and other interdisciplinary teams

**Figure 5.**
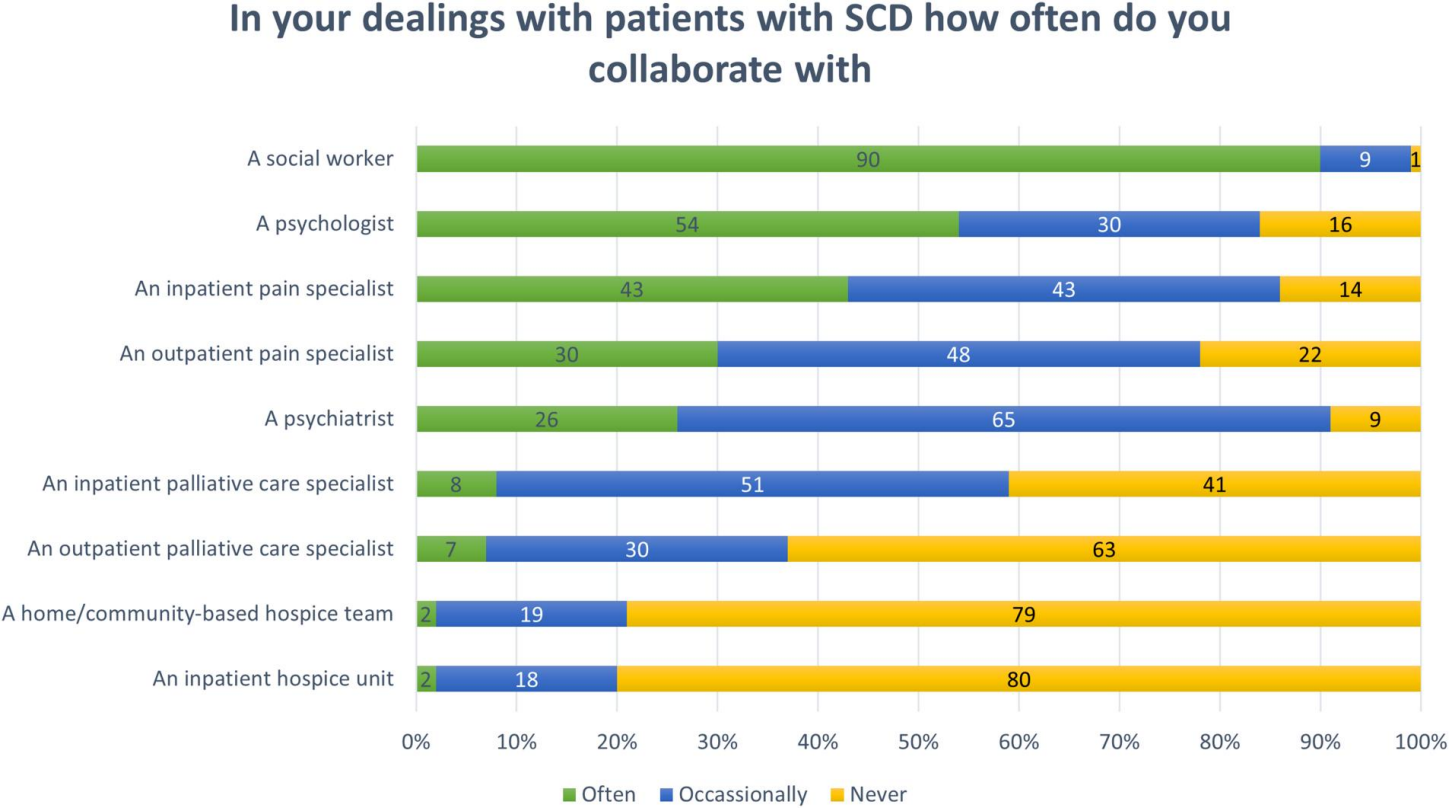
Participant responses to survey items regarding SCD clinician sense of ownership over PC interventions for SCD. “Agree” and “strongly agree” responses were combined. “Disagree” and “strongly disagree” responses were combined. The values represent percent of participant responses.

More than half of participants (59%) reported frequent or occasional collaboration with inpatient PC specialists. Most participants reported that they never collaborate with outpatient PC specialists (63%), home-based or community-based hospice teams (79%), or inpatient hospice teams (80%). Most participants reported frequent collaboration with psychiatrists, psychologists, social workers, and pain specialists (Figure 5).

### Clinician perceptions of patient reactions to palliative care referral

**Figure 6.**
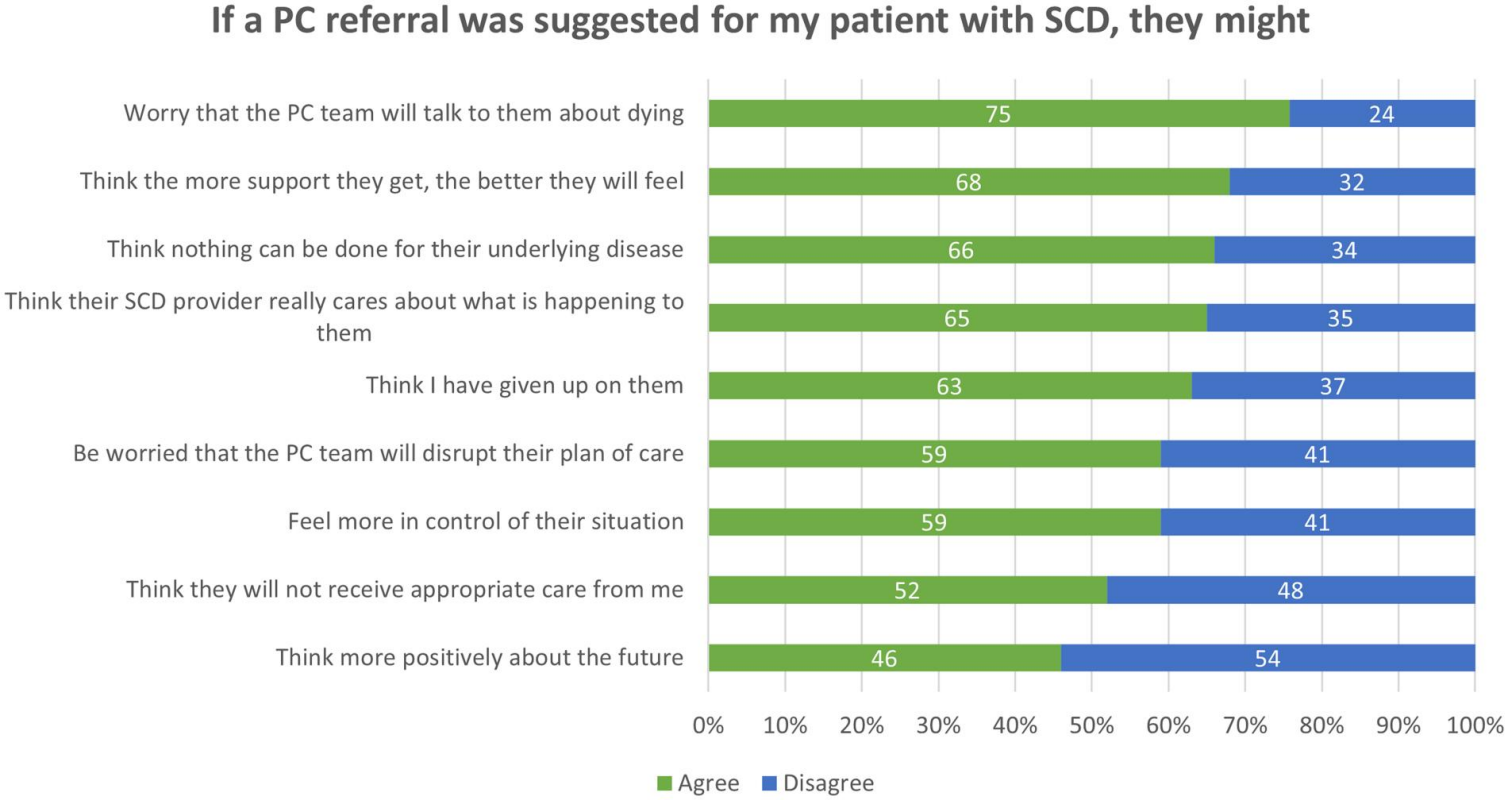
Participant responses to survey items regarding SCD clinician perceptions of patient reactions to PC referral. “Agree” and “strongly agree” responses were combined. “Disagree” and “strongly disagree” responses were combined. The values represent percent of participant responses.

Participant responses to items regarding clinician perceptions of how patients would react to a PC referral are presented in Figure 6. Most participants agreed that the patient might worry the PC specialist will talk to them about dying (76%), think nothing can be done for their disease (67%), and think their primary SCD clinician gave up on them (63%). Most participants agreed that their patients would feel in greater control of their situation (59%) and that their SCD clinician cares about what is happening to them (65%) if referred to PC. Then, we examined differences in clinician perceptions related to patient responses to PC referral stratified by clinician demographics (Table 3A–C). This analysis revealed a significant result: a pattern of different clinician perceptions was related to years of clinical practice. Clinicians with less than five years of clinical experience (100%) agreed that patients might worry PC teams will discuss dying and believe that nothing can be done for their condition, compared to the attitudes of clinicians with 5-14 years of clinical experience (73%) or more than 15 years of clinical experience (69%).

**Table 3.** Differences in percent agreement with survey items regarding SCD clinician attitudes toward palliative care, stratified by demographic characteristics.

A						
	Worry that PC team will talk to them about dying (*n *= 65, 75.58%)		Think the more support they get the better they will feel (*n *= 58, 67.44%)		Think nothing can be done for their underlying disease (*n *= 57, 66.28%)	
	*N* (%)	*P*-value^a^	*N* (%)	*P*-value^a^	*N* (%)	*P*-value^a^
**Sex**						
Cis-Female (*n *= 62)	46 (74%)	.77	44 (71%)	.18	43 (69%)	.42
Cis-Male (*n *= 21)	17 (81%)		11 (52%)		12 (57%)	
**Medical specialty**						
Adult hematology (*n *= 35)	27 (77%)	1.00	64 (69%)	1.00	21 (60%)	.12
Pediatric hematology (*n *= 38)	29 (76%)		26 (68%)		30 (79%)	
**Years of clinical practice**						
<5 years (*n *= 14)	14 (100%)	**.04**	10 (71%)	.34	13 (93%)	.053
5–14 years (*n *= 33)	24 (74%)		25 (76%)		19 (58%)	
≥15 years (*n *= 39)	27 (69%)		23 (59%)		25 (64%)	
**Prior palliative care training**						
No (*n *= 49)	38 (78%)	.80	36 (73%)	.25	33 (67%)	.82
Yes (*n *= 37)	27 (73%)		22 (59%)		24 (65%)	
**Practice type**						
Teaching hospital (*n *= 40)	28 (70%)	.10	30 (75%)	.10	26 (65%)	.63
Comp SCD center (*n *= 38)	33 (87%)		21 (55%)		27 (71%)	
**Prior referral to palliative care**						
No (*n *= 35)	26 (74%)	1.00	25 (71%)	.64	25 (71%)	.49
Yes (*n *= 51)	39 (76%)		33 (65%)		32 (63%)	


B						
	Think the SCD provider really cares about what is happening to them (total *n *= 56, 65.12%)		Think I have given up on them (total *n *= 54, 62.79%)		Be worried that the PC team will disrupt their plan of care (*n *= 50, 58.14%)	
	*N* (%)	*P*-value^a^	*N* (%)	*P*-value^a^	*N* (%)	*P*-value^a^
**Sex**						
Cis-Female (*n *= 62)	39 (63%)	.80	42 (68%)	.43	38 (61%)	.80
Cis-Male (*n *= 21)	14 (67%)		12 (57%)		12 (57%)	
**Medical specialty**						
Adult hematology (*n *= 35)	20 (57%)	.23	23 (66%)	1.00	21 (60%)	1.00
Pediatric hematology (*n *= 38)	27 (71%)		25 (66%)		22 (58%)	
**Years of clinical practice**						
<5 years (*n *= 14)	9 (64%)	.24	9 (64%)	.87	9 (64%)	.77
5–14 years (*n *= 33)	25 (76%)		22 (67%)		20 (61%)	
≥15 years (*n *= 39)	22 (56%)		23 (59%)		21 (54%)	
**Prior palliative care training**						
No (*n *= 49)	31 (63%)	.82	34 (69%)	.18	31 (63%)	.28
Yes (*n *= 37)	25 (68%)		20 (54%)		19 (51%)	
**Practice type**						
Teaching hospital (*n *= 40)	25 (63%)	.82	23 (58%)	.16	21 (53%)	.26
Comp SCD center (*n *= 38)	25 (66%)		28 (74%)		25 (66%)	
**Prior referral to palliative care**						
No (*n *= 35)	22 (63%)	.82	23 (66%)	.66	21 (60%)	.83
Yes (*n *= 51)	34 (67%)		31 (61%)		29 (57%)	

C						
	Feel more in control of their situation (total *n *= 51, 59.30%)		Think they will not receive appropriate care from me (*n *= 45, 52.33%)		Think more positively about the future (*n *= 40, 46.51%)	
	*N* (%)	*P*-value^a^	*N* (%)	*P*-value^a^	*N* (%)	*P*-value^a^
**Sex**						
Cis-Female (*n *= 62)	37 (60%)	0.61	34 (55%)	.62	29 (47%)	.61
Cis-Male (*n *= 21)	11 (52%)		10 (48%)		8 (38%)	
**Medical specialty**						
Adult hematology (*n *= 35)	18 (51%)	0.35	23 (66%)	.10	14 (40%)	.48
Pediatric hematology (*n *= 38)	24 (63%)		17 (45%)		19 (50%)	
**Years of clinical practice**						
<5 years (*n *= 14)	9 (64%)	0.18	9 (64%)	.65	8 (57%)	.08
5–14 years (*n *= 33)	23 (70%)		17 (52%)		19 (58%)	
≥15 years (*n *= 39)	19 (49%)		19 (49%		13 (33%)	
**Prior palliative care training**						
No (*n *= 49)	28 (57%)	0.66	29 (59%)	.19	19 (39%)	.13
Yes (*n *= 37)	23 (62%)		16 (43%)		21 (57%)	
**Practice type**						
Teaching hospital (*n *= 40)	25 (63%)	0.49	19 (48%)	.27	21 (53%)	.12
Comp SCD center (*n *= 38)	20 (53%)		23 (61%)		13 (34%)	
**Prior referral to palliative care**						
No (*n *= 35)	19 (54%)	0.51	19 (54%)	.83	16 (46%)	1.00
Yes (*n *= 51)	32 (63%)		26 (51%)		24 (47%)	

The total number of participants who agreed (“agree” and “strongly agree” responses were combined) with the survey item is presented as *N* (%). Bold indicates statistically significant value.

a Fisher’s exact test was used to calculate *P*-values.

Abbreviation: Comp SCD center, comprehensive sickle cell center.

## DISCUSSION

This study showed that most clinicians agreed that patients with SCD would benefit from early PC intervention, and essentially all clinicians disagreed with the perception that PC is only for patients at the end of life. Clinicians reported that they frequently managed symptoms for their patients with SCD, whereas they were less involved in managing end-of-life interventions. Clinicians reported regular collaboration with specialist disciplines such as pain services and social workers, although collaboration with PC teams was less common. Some survey participants believed that PC clinicians lack sufficient understanding of SCD to effectively manage its symptoms. A significant percentage of clinicians believed that patients with SCD would have a negative perception of referral to PC specialists.

The involvement of PC specialists in SCD care teams has been limited, and is usually introduced only at the end of life. Our study highlights an urgent need for increased collaboration between SCD and PC clinicians to overcome the factors contributing to low PC usage in SCD, including the ambivalence of some SCD clinicians about PC clinicians’ knowledge of SCD symptoms and their ability to care for these patients. Increased collaboration between SCD and PC clinicians will foster bidirectional learning: SCD clinicians will learn about the comprehensive interventions that PC can provide at all stages of SCD, and PC clinicians will learn about SCD to provide evidence-based care. This collaboration will ensure that patients with SCD receive the best interventions for symptom management and holistic care.

Our study confirmed the high symptom burden associated with SCD, as SCD clinicians reported frequent involvement in managing physical and psychological symptoms and expressed a sense of ownership in treating these symptoms. Previous studies reported that patients with chronic conditions have unmet PC needs that significantly impact their quality of life, even in optimal care environments offering multidisciplinary support such as CF centers.^25^ This further emphasizes the need for collaboration between SCD and PC clinicians to manage symptoms in SCD. PC clinicians have expertise in symptom management and interdisciplinary care, and can provide valuable insights and recommendations for the care of patients with SCD and challenging or refractory symptoms. Collaboration between SCD and PC clinicians also can optimize end-of-life care in SCD. Our study showed that SCD clinicians are rarely involved in end-of-life treatment or recommendation of hospice transition. Few studies have investigated the end-of-life preferences and experiences of patients with SCD.^26^ Advance care planning (ACP) is a common tool used to elicit and record a patient’s end-of-life preferences.^27^^,^^28^ Prior studies indicate that patients with SCD generally respond positively to ACP when introduced in the appropriate setting by a trusted clinician.^29–31^ This underscores the importance of early integration of PC specialists during discussions about ACP and serious illness for patients with SCD. Early integration of PC teams fosters a foundation of trust, which can enhance discussions about end-of-life preferences and improved end-of-life experiences for patients and families.

Many SCD clinicians expressed concerns that PC referral might provoke negative thoughts in their patients, such as feelings of abandonment or the perception that their healthcare provider gave up on them. These concerns are consistent with the results from other studies.^22^^,^^32^ PC is often associated with hospice care, and these misconceptions have been well-documented in the literature.^33^^,^^34^ Other studies revealed that patients lack a comprehensive understanding of the full scope of PC interventions,^35^^,^^36^ and one study reported that 71% of adult participants had no knowledge of PC.^36^ Other studies consistently indicated that patients who have more knowledge about PC are more likely to have positive perceptions of PC. A national survey of hematopoietic stem cell transplant recipients demonstrated that patient perceptions of PC are more positive than those predicted by their physicians.^37^ Family members often express a willingness to recommend PC to their loved ones after it is clearly defined and discussed with them.^38^ These collective studies underscore a critical need for patient and provider education about the role and scope of PC interventions, particularly among patients with SCD. Further studies are needed to evaluate the understanding and perception of PC among patients with SCD. Improving patients’ understanding of PC may lead to more positive perceptions and informed decision-making regarding PC interventions.

Although this study is limited by a small sample size, the results provide important preliminary data that will support future research and clinical practice in PC referral for patients with SCD. The lack of a comprehensive national directory of all clinicians involved in SCD management suggests that additional eligible participants were not included in this study. The study design could lead to self-selection bias when potential participants with more positive perceptions of PC elect to respond to the survey compared to non-participants with ambivalent or less favorable perceptions of PC. Most of the study participants were physicians, with only a few advanced practice practitioners (APPs). This distribution does not accurately reflect the current healthcare workforce, which includes a significant number of APPs providing care for patients with SCD. Additionally, social workers were not included in this study, despite their important role in assessing and managing the psychosocial needs of patients with SCD. Our future studies are designed to be more inclusive of the interdisciplinary team members involved in the care of individuals with SCD.

Our future research will focus on understanding the perceptions of PC among patients with SCD and their caregivers. Additionally, we aim to explore PC clinicians’ comfort levels in managing individuals with SCD and their views on the role of PC involvement with this population. Together with the current study, these future studies will help us identify areas for intervention to enhance PC involvement in SCD. Similar initiatives have been undertaken to address unmet PC needs in CF, resulting in a consensus statement on models of PC delivery for individuals with CF.^39–41^ Likewise, the development of PC delivery models is needed for SCD.

## CONCLUSION

Clinicians treating patients with SCD had a positive attitude toward PC and supported the early integration of PC in sickle cell management. This exploratory study highlights the need for educational interventions to elevate knowledge about the comprehensive scope of PC interventions among SCD clinicians and patients. These efforts will demystify and destigmatize PC, thereby fostering greater collaboration among SCD and PC clinicians, particularly in outpatient settings. Integration of PC into cancer and heart failure management enhanced the quality of life for those patients, and could potentially enhance the quality of life for patients with SCD.

## Data Availability

Data used in this study can be obtained from the corresponding author upon reasonable request.

## References

[1] Hassell KL. Population estimates of sickle cell disease in the U.S. Am J Prev Med. 2010;38(4 Suppl):S512-21. 10.1016/j.amepre.2009.12.022 20331952

[2] Piel FB , Steinberg MH, Rees DC. Sickle cell disease. N Engl J Med. 2017;377(3):305. 10.1056/NEJMc1706325 28723338

[3] Blinder MA , Duh MS, Sasane M, Trahey A, Paley C, Vekeman F. Age-related emergency department reliance in patients with sickle cell disease. J Emerg Med. 2015;49(4):513-522.e1. 10.1016/j.jemermed.2014.12.080 25910824

[4] Chen M , Ataga KI, Hankins JS, et al Age-related differences in risks and outcomes of 30-day readmission in adults with sickle cell disease. Ann Hematol. 2023;102(9):2329-2342. 10.1007/s00277-023-05365-5 37450055

[5] Udeze C , Evans KA, Yang Y, et al Economic and clinical burden of managing sickle cell disease with recurrent vaso-occlusive crises in the United States. Adv Ther. 2023;40(8):3543-3558. 10.1007/s12325-023-02545-7 37332020 PMC10329958

[6] Hulihan M , Hassell KL, Raphael JL, Smith-Whitley K, Thorpe P. CDC grand rounds: improving the lives of persons with sickle cell disease. MMWR Morb Mortal Wkly Rep. 2017;66(46):1269-1271. 10.15585/mmwr.mm6646a2 29166365 PMC5769787

[7] Platt OS , Brambilla DJ, Rosse WF, et al Mortality in sickle cell disease. Life expectancy and risk factors for early death. N Engl J Med. 1994;330(23):1639-1644. 10.1056/NEJM199406093302303 7993409

[8] Johnston EE , Adesina OO, Alvarez E, et al Acute care utilization at end of life in sickle cell disease: highlighting the need for a palliative approach. J Palliat Med. 2020;23(1):24-32. 10.1089/jpm.2018.0649 31390292 PMC13175313

[9] Brandow AM , Carroll CP, Creary S, et al American Society of Hematology 2020 guidelines for sickle cell disease: management of acute and chronic pain. Blood Adv. 2020;4(12):2656-2701. 10.1182/bloodadvances.2020001851 PMC7322963 32559294

[10] Osunkwo I , O'Connor HF, Saah E. Optimizing the management of chronic pain in sickle cell disease. Hematology. 2020;2020(1):562-569. 10.1182/hematology.2020000143 33275672 PMC7727591

[11] Osunkwo I , Andemariam B, Minniti CP, et al Impact of sickle cell disease on patients’ daily lives, symptoms reported, and disease management strategies: Results from the international Sickle Cell World Assessment Survey (SWAY). Am J Hematol. 2021;96(4):404-417. 10.1002/ajh.26063 33264445 PMC8248107

[12] Levenson JL , McClish DK, Dahman BA, et al Depression and anxiety in adults with sickle cell disease: the PiSCES project. Psychosom Med. 2008;70(2):192-196. 10.1097/PSY.0b013e31815ff5c5 18158366

[13] Chang YK , Allen LA, McClung JA, et al Criteria for referral of patients with advanced heart failure for specialized palliative care. J Am Coll Cardiol. 2022;80(4):332-344. 10.1016/j.jacc.2022.04.057 35863850 PMC10615151

[14] Lee L , Smith-Whitley K, Banks S, Puckrein G. Reducing health care disparities in sickle cell disease: a review. Public Health Rep. 2019;134(6):599-607. 10.1177/0033354919881438 31600481 PMC6832089

[15] Kanter J , Smith WR, Desai PC, et al Building access to care in adult sickle cell disease: defining models of care, essential components, and economic aspects. Blood Adv. 2020;4(16):3804-3813. 10.1182/bloodadvances.2020001743 32785684 PMC7448595

[16] Dampier C , Lieff S, LeBeau P, et al Health-related quality of life in children with sickle cell disease: a report from the Comprehensive Sickle Cell Centers Clinical Trial Consortium. Pediatr Blood Cancer. 2010;55(3):485-494. 10.1002/pbc.22497 20658620 PMC2911637

[17] Dampier C , LeBeau P, Rhee S, et al Health-related quality of life in adults with sickle cell disease (SCD): a report from the comprehensive sickle cell centers clinical trial consortium. Am J Hematol. 2011;86(2):203-205. 10.1002/ajh.21905 21264908 PMC3554393

[18] Bakitas M , Lyons KD, Hegel MT, et al Effects of a palliative care intervention on clinical outcomes in patients with advanced cancer: the Project ENABLE II randomized controlled trial. JAMA. 2009;302(7):741-749. 10.1001/jama.2009.1198 19690306 PMC3657724

[19] Temel JS , Greer JA, Muzikansky A, et al Early palliative care for patients with metastatic non-small-cell lung cancer. N Engl J Med. 2010;363(8):733-742. 10.1056/NEJMoa1000678 20818875

[20] El-Jawahri A , Nelson AM, Gray TF, Lee SJ, LeBlanc TW. Palliative and end-of-life care for patients with hematologic malignancies. J Clin Oncol. 2020;38(9):944-953. 10.1200/JCO.18.02386 32023164 PMC8462532

[21] LeBlanc TW , Roeland EJ, El-Jawahri A. Early palliative care for patients with hematologic malignancies: is it really so difficult to achieve? Curr Hematol Malig Rep. 2017;12(4):300-308. 10.1007/s11899-017-0392-z 28639084

[22] El-Jawahri A , LeBlanc TW, Burns LJ, et al What do transplant physicians think about palliative care? A national survey study. What do transplant physicians think about palliative care? A National Survey Study. Cancer. 2018;124(23):4556-4566. 10.1002/cncr.31709[PMC][30289980] 30289980 PMC6289734

[23] Hui D , Park M, Liu D, Reddy A, Dalal S, Bruera E. Attitudes and beliefs toward supportive and palliative care referral among hematologic and solid tumor oncology specialists. Oncologist. 2015;20(11):1326-1332. 10.1634/theoncologist.2015-0240 26417037 PMC4718432

[24] Nwogu-Onyemkpa E , Dongarwar D, Salihu HM, et al Inpatient palliative care use by patients with sickle cell disease: a retrospective cross-sectional study. BMJ Open. 2022;12(8):e057361. 10.1136/bmjopen-2021-057361 PMC9386219 35973707

[25] Trandel ET , Pilewski JM, Dellon EP, et al Prevalence of unmet palliative care needs in adults with cystic fibrosis. J Cyst Fibros. 2020;19(3):394-401. 10.1016/j.jcf.2019.11.010 31862306 PMC8662333

[26] Bartholomew F , De Castro LM. Advance care planning in adults with sickle cell disease (SCD). Blood. 2010;116(21):391-391. 10.1182/blood.V116.21.391.391

[27] Bischoff KE , Sudore R, Miao Y, Boscardin WJ, Smith AK. Advance care planning and the quality of end-of-life care in older adults. J Am Geriatr Soc. 2013;61(2):209-214. 10.1111/jgs.12105 23350921 PMC3760679

[28] Levoy K , Sullivan SS, Chittams J, Myers RL, Hickman SE, Meghani SH. Don’t throw the baby out with the bathwater: meta-analysis of advance care planning and end-of-life cancer care. J Pain Symptom Manage. 2023;65(6):e715-e743. 10.1016/j.jpainsymman.2023.02.003 36764411 PMC10192153

[29] Oyedeji CI , Oyesanya T, Gray N, Strouse JJ. Barriers and facilitators of advance care planning for older adults with sickle cell disease. Blood. 2020;136(Supplement 1):58-59. 10.1182/blood-2020-136231

[30] Oyedeji CI , Strouse JJ, Masese R, Gray N, Oyesanya TO. "Death is as Much Part of Life as Living": Attitudes and experiences preparing for death from older adults with sickle cell disease. Omega (Westport). 20;90(3):1056-1077. 10.1177/00302228221116513 35857485 PMC10082645

[31] Ramsey CP. Young Adult African American family members’ perceptions, knowledge, attitudes, and utilization toward advance directives. ABNF J Spring. 2013;24(2):51-59. 23734473

[32] Ufere NN , Donlan J, Waldman L, et al Physicians’ perspectives on palliative care for patients with end-stage liver disease: A National Survey Study. Liver Transpl. 2019;25(6):859-869. 10.1002/lt.25469 30963669 PMC6529275

[33] Ramos K , Kaufman BG, Winger JG, et al Knowledge, goals, and misperceptions about palliative care in adults with chronic disease or cancer. Palliat Support Care. 2024;22(6):1707-1713. 10.1017/S1478951523001141 PMC10858297 37559194

[34] Zimmermann C , Swami N, Krzyzanowska M, et al Perceptions of palliative care among patients with advanced cancer and their caregivers. CMAJ. 2016;188(10):E217-E227. 10.1503/cmaj.151171 27091801 PMC4938707

[35] Langan E , Kamal AH, Miller KEM, Kaufman BG. Comparing palliative care knowledge in metropolitan and nonmetropolitan areas of the United States: Results from a National Survey. J Palliat Med. 2021;24(12):1833-1839. 10.1089/jpm.2021.0114 34061644

[36] Huo J , Hong YR, Grewal R, et al Knowledge of palliative care among American Adults: 2018 Health Information National Trends Survey. J Pain Symptom Manage. 2019;58(1):39-47 e3. 10.1016/j.jpainsymman.2019.03.014 30922703

[37] Barata A , Abrams HR, Meyer C, et al What do patients think about palliative care? A national survey of hematopoietic stem cell transplant recipients. Blood Adv. 2023;7(10):2032-2041. 10.1182/bloodadvances.2023009712 36877661 PMC10188629

[38] Shalev A , Phongtankuel V, Kozlov E, Shen MJ, Adelman RD, Reid MC. Awareness and misperceptions of hospice and palliative care: A population-based survey study. Am J Hosp Palliat Care. 2018;35(3):431-439. 10.1177/1049909117715215 28631493 PMC5866727

[39] Dellon EP , Basile M, Hobler MR, et al Palliative care needs of individuals with cystic fibrosis: perspectives of multiple stakeholders. J Palliat Med. 2020;23(7):957-963. 10.1089/jpm.2019.0464 32023421 PMC8255308

[40] Dellon EP , Helms SW, Hailey CE, et al Exploring knowledge and perceptions of palliative care to inform integration of palliative care education into cystic fibrosis care. Pediatr Pulmonol. 2018;53(9):1218-1224. 10.1002/ppul.24073 29862668

[41] Kavalieratos D , Georgiopoulos AM, Dhingra L, et al Models of palliative care delivery for individuals with cystic fibrosis: cystic fibrosis foundation evidence-informed consensus guidelines. J Palliat Med. 2021;24(1):18-30. 10.1089/jpm.2020.0311 32936045 PMC7757696

